# Living with phenylketonuria in adulthood: The PKU ATTITUDE study

**DOI:** 10.1016/j.ymgmr.2018.06.007

**Published:** 2018-07-11

**Authors:** Chiara Cazzorla, Giulia Bensi, Giacomo Biasucci, Vincenzo Leuzzi, Filippo Manti, Antonella Musumeci, Francesco Papadia, Vera Stoppioni, Albina Tummolo, Marcella Vendemiale, Giulia Polo, Alberto Burlina

**Affiliations:** a Division of Inherited Metabolic Diseases, Reference Centre Expanded Newborn Screening, Department of Woman's and Child's Health - University Hospital, Padova, Italy; b Department of Pediatrics and Neonatology, Regional Referral Clinical Centre for IMD, Guglielmo da Saliceto Hospital, Piacenza, Italy; c Department of Human Neuroscience, Child Neurology and Psychiatry - Sapienza University, Rome, Italy; d Division of Child Neurology and Psychiatry, Riuniti Hospital Marche Nord Pesaro, Fano, Italy; e Department of Metabolic Diseases, Clinical Genetics and Diabetology, Giovanni XXIII Children's Hospital, Bari, Italy

**Keywords:** Phenylketonuria (PKU), Diet, Adulthood, Compliance, Amino acid

## Abstract

Dietary treatment is the cornerstone of therapy for phenylketonuria (PKU), but adherence to low- phenylalanine diet progressively decreases after adolescence. We designed a survey to characterize the dietary habits of Italian adult PKU patients and to identify psychological factors influencing disease perception and adherence to diet. Participants to the survey (*n* = 111; response rate 94%) were asked to complete a structured questionnaire. Patients appeared to have an altered perception and awareness of the disease. About 40% of them did not consider PKU a disease and, despite declaring regular monitoring of phenylalanine levels (85%), nearly half of them reported a high plasma value over the last 6 months (>600 μmol/L, 48%) or were unable to specify it (31%). Adherence to PKU diet was unsatisfactory, with increased consumption of natural protein sources and reduced daily use of amino-acid supplements (<4–5 times/day in 82% patients). In addition to the intrinsic characteristics of AA formula (palatability, ease of use), the most important factor influencing their consumption was the increased social pressure associated with their use (55%). Plasma phenylalanine periodical measurements (61%) and examinations at metabolic centers (49%) were considered relevant for compliance to diet. In Italian adult PKU patients dietary management was found to be inadequate, likely due to inappropriate perception and knowledge of the disease, and lack of awareness of the negative impact of poor metabolic control in adult life. Clinicians should consider implementing more intense and tailored educational measures, as well as structured transitional care processes.

## 1 Introduction

Phenylketonuria (PKU) is an autosomal recessive metabolic disorder that affects about one person every 10,000 births in Europe [1]. PKU is determined by the impairment of phenylalanine hydroxylase (PAH) activity resulting in decreased phenylalanine (PHE) conversion to tyrosine. Deficiency of the hepatic PAH results in a broad spectrum of hyperphenylalaninemia (HPA) ranging from very mild HPA (blood PHE: 120–600 μmol/L), to mild PKU (blood PHE: 600–1200 μmol/L) and classic PKU (blood PHE > 1200 μmol/L) [2]. Accordingly, the alteration leads to high plasma concentrations of phenylalanine (and decreased tyrosine), which accumulates in the tissues and causes damage if left untreated. The most serious consequence is the impaired development of the central nervous system, with intellectual disability frequently being associated with other manifestations, such as motor disturbances, psychiatric symptoms, aberrant behavior, and epilepsy [3]. Low-PHE diet (a personalized dietary plan based on the consumption of normal food sources, low-protein foods and the use PHE-free protein substitutes to cover protein requirements) is the mainstay of treatment of PKU [4]. During childhood adherence to diet is very high, but progressively decreases after adolescence [[5], [6], [7]]. This is likely due to increasing independency of patients from the family as well as to psychological and social burden both in patients and their families [8, 9].

Dietary management in childhood is associated with neurocognitive outcome [[10], [11], [12]]. Individuals with early-treated PKU can achieve normal or near-normal IQ if they maintain good metabolic control through dietary restriction until the age of 12, but even after this age there is a correlation between current phenylalanine levels and IQ among individuals aged between 0 and 39 years [13]. PHE levels may still influence the adolescent and adult brain anyway, and patients with elevated PHE levels have been reported to show neuroradiological [14] and neuropsychological deficits, as well as impairment of executive function, speed and attention [15, 16]. In adult PKU patients (age > 32 years) poor cognitive performance, assessed by IQ and information processing, was found to correlate with higher blood PHE levels in adolescence, possibly related to early relaxation of diet at age 10 [15]. Besides, neuroimaging structural alterations in the white matter have been reported in adult PKU patients with insufficient metabolic control, although the lesions do not appear to be associated with clinical symptoms [11, 16, 17]. As there is currently no strong evidence that it is safe to discontinue dietary treatment in adults, lifelong treatment is recommended, even though it is acknowledged that dietary management is associated with significant patient burden [3, 18]. In recent years, the focus has increasingly shifted to the Quality of Life (QoL) of PKU patients previously receiving dietary intervention. Although QoL scores of PKU patients are reported to be generally similar to healthy controls, a chronic disease can engender anxiety and fear of not being able to adequately control the course of the disease [19].

Reviews have recently pointed out that there is a paucity of studies examining the influence of demographic and psychological factors on metabolic control in adult PKU patients [8, 20]. Moreover, no studies have addressed the issue from the point of view of the patient. The aim of this multicenter survey (Analysis of the mosT relevanT and predIctive facTors inflUencing aDherence to PKU diEt [ATTITUDE]) was to collect information on the subjective perceptions of the patients by carrying out in-depth interviews of Italian adult PKU patients.

## 2 Materials and methods

Over a 12 month period (from August 2016 to August 2017) 116 adult patients were recruited for the study. Patient inclusion criteria for enrollment were: confirmed diagnosis of PKU by neonatal screening, age ≥ 16 years, treatment with a PHE-restricted diet from birth and/or tetrahydrobiopterin (BH4) and IQ > 70.

A total of 116 eligible patients were contacted: 111 (97%) were selected to participate in the study, considering gender, disease severity and age criteria; reasons of exclusion were: refusal to participate (*n* = 4), and recent childbirth (*n* = 1).

In our sample, 88 patients were affected by the classical form and 23 were affected by the mild form. A total of 92 patients were on PHE restricted diet only; 19 patients over the last 4 years were found to be BH4 responsive and therefore therapy was switched from dietary treatment alone to BH4 treatment.

The study was carried out at five different Italian centers (listed among authors' affiliations) distributed throughout the country (North = 2; Centre = 2; South = 1). Except for one center (Division of Inherited metabolic diseases of Padova) all other patients were followed by pediatricians or pediatric neurologists. These centers continue to care for PKU patients even after they become adults. Transition program is available in very few centers in Italy. Furthermore, these centers were chosen because their clinical team included a dedicated psychologist.-.

The survey was developed based on a survey that was already used for other chronic diseases, such as diabetes, and modified for PKU. It has been already used in a single-center (Padua) pilot study. Responders were asked to complete a two-section survey. The first part of the survey addressed socio-demographic (family, school or work environment, leisure time) and general clinical data, as well as disease-related symptoms. Then, patients prescribed a low-PHE nutritional plan only were asked to complete the second part of the survey, which investigated dietary habits and psychological factors influencing adherence to diet more in depth. Standard operating methodology was achieved before data collection by organizing a training session with all involved healthcare professionals. At each center, all patients were evaluated by the same medical doctor and psychologist. Specifically, the former was responsible for survey explanation, written informed consent and clinical examination, while the latter administered the survey.

The study was performed in compliance with local regulatory requirements and written informed consent was obtained from all subjects or their legally authorized representatives.

### 2.1 Statistical analysis

Descriptive statistical analyses were carried out. Categorical variables were presented as counts and percentages, while continuous variables were reported as mean and standard deviation (normal distribution) or median and inter-quartile range (IQR; non-normal distribution). Categorical variables were compared using Fisher's exact test. All statistical analyses were performed using SAS® software (SAS Institute Inc., Cary, NC, USA).

## 3 Results

A total of 111 patients answered the survey across the 5 metabolic centers (Bari: 35, Padua: 34, Fano: 18, Rome: 16 and Piacenza: 8). Regarding demographic features of the study population, these were similar across recruiting centers: overall, 61 females (55%) and 50 males (45%); age range 19–30 years, mean 24 years old.

The first part of the survey was completed by all patients (*n* = 111), while results regarding dietary habits and psychological factors influencing adherence to diet refer to patients not taking BH4 and following a dietary prescription only (*n* = 92).

Information on family, school or work environment, and leisure time is reported in Table 1. Data appeared to be mainly in agreement with those of the general Italian population [22]. Seven patients out of 111 (6.3%) claimed to study and work at the same time, while 17 patients (15.3%) were unemployed.

**Table 1:** Information on family, school or work environment, and leisure time (*N* = 111).

Question	N (%)
Category B driving license (YES)	83 (84.6)⁎
Who are you living with? *- Alone* *- With my family*	14 (12.6) 97 (87.4)
Are you married or do you have a partner? (YES)	54 (48.6) (55.1)⁎
Please indicate your father's highest qualification in terms of education: *- primary or junior high school leaving certificate (8 years of education)* *- high school leaving certificate (13 years of education)* *- University Bachelor's degree or postgraduate diploma (≥16 years of* *education)*	63 (56.8) 37 (33.3) 11 (9.9)
Please indicate your mother's highest qualification in terms of education: *- primary or junior high school leaving certificate (8 years of education)* *- high school leaving certificate (13 years of education)* *- University Bachelor's degree or postgraduate diploma (≥16 years of* *education)*	74 (66.7) 30 (27.0) 7 (6.3)
Are you attending school? (YES) - *high school (13 years of education)* *- University (≥16 years of education)* *- Not specified*	39 (35.1) 24 14 1
Are you currently working? (YES)	62 (55.9)
Does your work involve travelling? (YES)	12 (10.8)
Do you practice sports? (YES)	58 (52.3)

⁎ Calculated on those aged ≥18 years (*n* = 98).

**Fig. 1:**
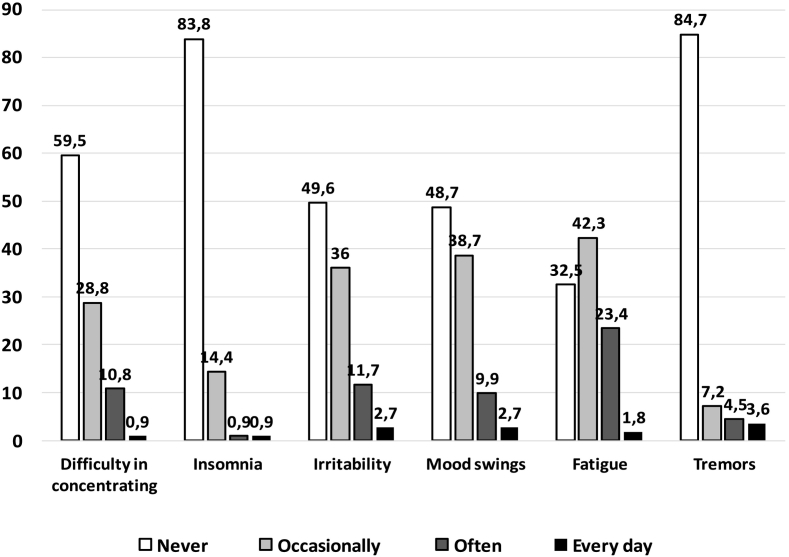
Frequency (%) of main symptoms that could be ascribed to high plasma PHE levels in the overall study population.

A total of 46 patients (40%) did not consider PKU a disease. However, symptoms that could be ascribed to high plasma PHE levels [8, 23] were reported often (at least twice a week) or even every day in a significant proportion of patients (Fig. 1), particularly fatigue (*n* = 28; 25%), irritability (*n* = 16; 14%), mood swings (*n* = 14; 13%) and difficulty in concentrating (*n* = 13; 12%).

The number of attended examinations over the last 2 years was in agreement with current guideline-based recommendations (57 patients reported 2 examinations over the last 2 years) [4]. In addition, regular monitoring of PHE plasma levels was reported by most patients (*n* = 94; 85%) (Table 2). However, an acceptable maximum PHE value (<600 μmol/L) over the last 6 months was reported by less than one fourth of the patients (*n* = 24; 22%) while about one third (*n* = 34; 31%) did not specify it. In respect to this, there are no differences between patients on low PHE diet and patients taking BH4.

**Table 2:** Clinical/metabolic features and dietary habits of the study population.

Question	Overall (n = 111)	On low-PHE diet (n = 92)
Please indicate the number of medical examinations you have attended over the last 2 years, median [IQR]	2 [2–3]	2 [2–3]
Do you monitor phenylalanine plasma levels regularly? (YES)	94 (84.7)	81 (88)
What was your maximum value of phenylalanine over the last 6 months? - ≤600 μmol/L - 601-1000 μmol/L - >1000 μmol/L - not reported	24 (21.6) 32 (28.8) 21 (18.9) 34 (30.7)	22 (23.9) 25 (27.2) 20 (21.7) 25 (27.2)
Do you take tetrahydrobiopterin (BH4)? (YES)	19 (17.2)	0 (0.0)
Do you adhere to a low-phenylalanine diet? (YES)	92 (82.8)	92 (100)
Do you have lunch or dinner out? (YES)	–	74 (80.4)
If you do, how often on average in a week? Mean [SD]	–	2.6 [1.8]⁎
Please indicate the number of times you consume an amino acid mixture daily Mean [SD]	--	2.4 [1.3]
Do you have problems in taking your amino acid mixtures with you while travelling? (YES)	--	9 (9.8)
Please indicate whether you consume low protein foods - never - sometimes (at least twice a week) - habitually (at least once daily)	– – --	4 (4.3) 8 (8.6) 80 (87.1)
According to you, to what extent do you follow your PKU diet? (Completely)	--	39 (42.4)

Data are reported as count and percentage (between parentheses) unless otherwise reported.

⁎ Calculated on those reporting to have lunch or dinner out (n = 74).

**Fig. 2:**
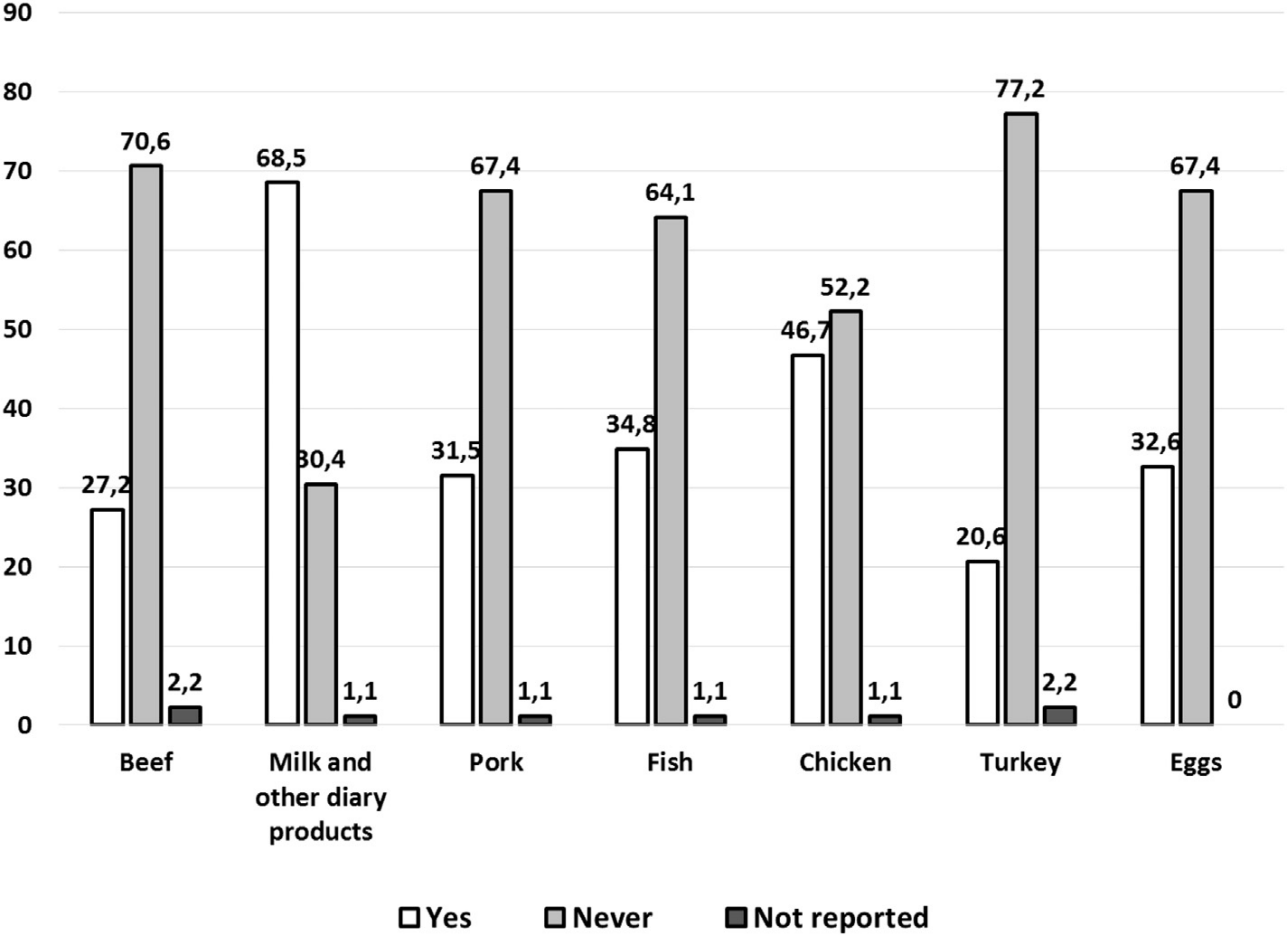
Prevalence of patients reporting the consumption of protein-containing foods.

Regarding information on dietary habits, 74 patients (80%) reported to generally have lunch or dinner out of home more than twice per week and 39 patients (42%) reported to completely follow a low-PHE nutritional plan. In respect to this, the habitual consumption (at least once daily) of low-protein foods was reported by most patients (*n* = 80; 87%), while the mean daily use of amino acid (AA) mixtures was rather low, with 17 patients (18.5%) reporting an optimal consumption (4–5 times/day) [4]. Natural sources of protein were consumed frequently (Fig. 2), particularly milk and other dairy products (63 patients reported up to 7 times/week). The frequency of consumption of other protein sources was usually 1–3 times/week.

**Fig. 3:**
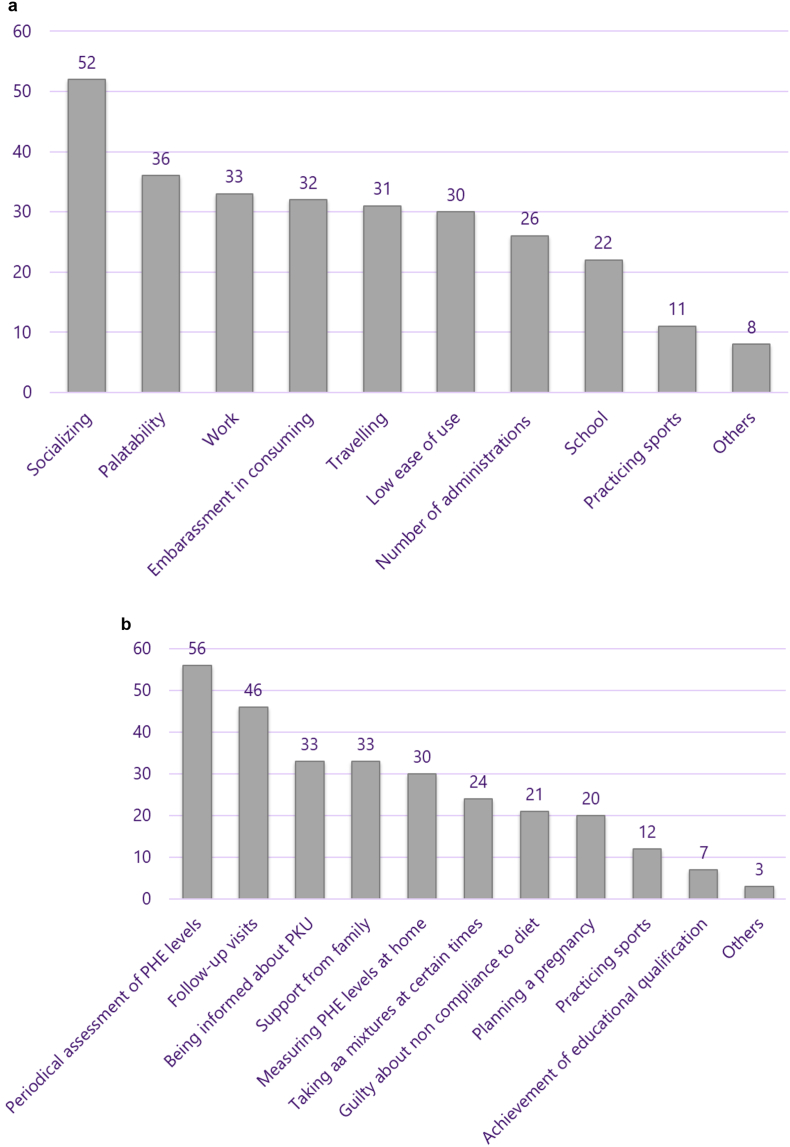
Factors that were reported to interfere the most with adherence to the consumption of amino acid mixtures (numbers of patients reporting yes or no, *Plot A*) and the nutritional plan for PKU (numbers of patients reporting yes or no) *Plot B*).

Finally, we investigated factors influencing adherence to diet. We asked which factors interfere the most with adherence to the consumption of AA mixtures: the most relevant factors were the impact of use on socializing (*n* = 52; 56%), palatability (*n* = 36; 39%), consumption in a working environment (*n* = 33; 36%), embarrassment in consuming mixtures when out of home (*n* = 32; 35%), travelling (*n* = 31; 34%) and low ease of use (*n* = 30; 33%). (Fig. 3, *Plot A*). Regarding factors that promote compliance with the nutritional plan, plasma PHE periodical measurements (*n* = 56; 61%), follow-up examinations at the metabolic center (*n* = 46; 50%), being informed about the disease (n = 33; 36%) and support from family (n = 33; 36%) – were indicated as the most important (Fig. 3, *Plot B*). For factors influencing both the use of AA mixtures and compliance to PKU diet, no significant differences between genders were observed with the exception of planning a pregnancy, which increased adherence to diet.

## 4 Discussion

Dietary treatment is the mainstay of PKU management and should be continued for life [2]. Unfortunately, this proves to be inescapably arduous for adults, given the sheer scale of the required reduction in dietary protein and the centrality of food to our cultural identities and social relationships.

To our knowledge, this is the first Italian survey carried out on adult patients diagnosed with PKU at birth, who have attended the same metabolic clinic ever since. What is more, our survey focused on the subjective perceptions of the patients instead of clinical data collected by healthcare professionals, since the perceptions of the former do not necessarily coincide with the perceptions of the latter. The survey has thus provided unique information on how patients actually experience PKU and related dietary treatment. The main finding was that compliance with dietary recommendations among adult PKU patients was poor, with less than half (42%) claiming full adherence, increased consumption of natural protein sources and reduced daily use of amino-acid supplements (<4–5 times/day in 82% patients). Their claims were supported by phenylalanine plasma concentration data: nearly half of them reported a high plasma value over the last 6 months (>600 μmol/L, 48%) or were unable to specify it (31%). This is consistent with the recent findings of the systematic review by Medford (2017) including 29 articles representing 1784 PKU patients of all ages, who found that the main factor associated with worsening of metabolic control was age [9], as well as a less recent retrospective chart review related to 125 PKU patients that showed that the proportion of adequately controlled subjects diminished considerably after the age of 18 (39% vs 57% between the age of 6 and 17 years) [24] and general claims in reviews on PKU in the literature [25]. Increasing age usually involves the transition from a pediatric to an adult setting. Even at a center that managed the transition successfully, not all adult PKU patients were adequately controlled [26]. Also the failure to find differences between genders is consistent with previous findings [9, 24].

The issue then becomes how important metabolic control is in adult PKU patients. The European guidelines have recommended treatment for life, with the exception of patients ≥12 years with untreated phenylalanine levels <600 μmol/l [2]. A recent nested, case-controlled study using insurance claims databases [27], in which patients were subdivided by age bracket, has shown that the prevalence of neuropsychiatric conditions is higher in PKU patients than in the general population, independently of age. In the overall PKU patient population (*n* = 3714) the prevalence of the following conditions was significantly (p<0.05) higher and at least 4 times the prevalence in the general population: intellectual disability (Prevalence Ratio (PR) 7.9), autism spectrum disorder (PR 6.1), Tourette / tic disorders (PR 5.4) and eating disorders (PR 4.0). In the 20–39 year age bracket i.e. the one that resembled our patient population the most (*n* = 2242), the prevalence of fatigue and attention disorders – issues that were reported by our interviewees - were also significantly more common (PR 2.2 and 1.7, respectively, both p<0.05). Our interviewees also reported mood swings, which have been found to be associated with phenylalanine concentrations (*p* = .039) [28].

Most of the factors impacting compliance with dietary treatment by our interviewees had already been identified for inherited metabolic diseases that required low-protein diet years ago. McDonald (2012) [25] subdivided them into clusters: those related to AA supplements (including two of the reasons listed i.e. lack of palatability and ease of use), burden of diet (including the risk of social isolation that covers the other three factors mentioned i.e. interference with socializing, difficulty in working environment and embarrassment) and four additional ones, namely the paradox of dietary treatment i.e. the lack of apparent correlation between well-being and dietary adherence, patient responsibility (including motivation, knowledge of treatment, development of relevant skills required for self-care), family characteristics (socio-economic status, cultural and religious factors, source of emotional support) and information.

The first of the additional four factors – the paradox – resulted to be very important: nearly one half of the interviewees (40%) did not consider PKU a disease and this lack of awareness could affect adherence to dietary recommendations. Indeed, additional multivariate analysis are necessary in order to highlight the possible correlation between variables.

Also the second one - level of patient responsibility - applies here. The American Academy of Pediatrics [29] recommends great care in ensuring a well-timed and structured transition from child- to adult oriented health care for any kind of chronic disease between the ages of 18 and 21 years. The Academy has developed an algorithm designed to ensure coordination of patient, family and healthcare professional responsibilities and ensure that young adults are adequately empowered so that they are in a position to take responsibility for self-care as an adult. In a recent study on the adequacy of knowledge on the disease and dietary requirements in 173 PKU patients aged 10–19 and 110 parents of PKU children [30], 98% parents claimed to teach their children to be self-reliant, but only 81% of the children confirmed that this was true. What is worse, only 45% (*n* = 74) of PKU patients knew daily PHE intake recommendations and only 27% of patients (*n* = 41) knew the PHE content in a minimum of three out of four researched food products. However, in this study lack of compliance was not just due to ignorance, since 30% of patients reported feeling very ashamed about their dietary restrictions and this appeared to induce them to have a negative attitude towards dietary treatment.

The other two additional factors – family characteristics in terms of socio-economic status and the level of education - have been found to be closely correlated with adequate metabolic control in another, much more common metabolic disorder, namely diabetes mellitus. A cross-sectional, multicenter study, in 768 subjects with type 1 diabetes under 18 years of age [31] showed that metabolic control was very poor (target HbAc1 values were achieved in only 28.1% of subjects) and was significantly correlated to these two factors (*p* = .02 for both factors).

Studies in PKU on the importance of the socio-economic status of the family have yielded inconsistent results [9]. The only common feature related to poor metabolic control that has been consistently found has been having divorced parents, likely because this indicates a lower level of family support [32], one of the positive factors mentioned by our interviewees.

Although education has been mentioned as one of the factors influencing adherence to dietary treatment by PKU patients, our survey showed the only one third of patients endorsed this item. A possible explanation of this controversial point is the difference between theory and real life.However, although this factor is necessary, it is not sufficient to guarantee adherence, since other psychological issues interfere with motivation [9, 30]. This is where follow-up visits come in. Indeed, they were listed among the important factors promoting compliance and have proved to be the key to successful outcome in clinical practice [26].

We acknowledge that our study is limited by its relatively small sample size (*n* = 111) reduced even further by the removal of results on patients treated only with BH4 (N pts. on restricted diet =92). The reason for the small sample size was not only the rarity of PKU, but also the fact that the interview required a psychologist with experience with PKU patients, who was a regular member of the clinical team. This requirement was met by a network of 5 centers in Italy. The adult PKU subgroup on treatment with BH4 was so small (*n* = 19) that we could not use the data to reach any conclusions. Results among this small group of patients highlighted that metabolic control may be not optimal in these patients also, therefore patients should be carefully monitored and therapy evaluated for its appropriateness. Further studies are required to address the adequacy of treatment in PKU adult patients belonging to this subgroup.

In conclusion, we have ascertained that metabolic control and compliance with dietary treatment in adult PKU patients are poor. The main factors affecting compliance are lack of awareness of the consequences, since some patients do not even consider a disease, psychological difficulties in coping with dietary restrictions in society and intrinsic negative features of AA supplements, whereas follow-up visits and family support promote compliance.

These factors need to be addressed taking a series of specific measures: setting up educational initiatives with the objective of increasing awareness about the condition and the need for lifelong dietary treatment; ensuring regular follow-up, which is to include structured transition from adolescence to adulthood and interventions by a psychologist, as appropriate; making targeted interventions on dietary treatment in the attempt to make it more acceptable, thus reducing its burden in daily life.

Further studies should focus on patients aged between 10 and 14, in order to identify factors (e.g psychological or dietary) associated with compliance to low-PHE diet. Increased awareness on the importance of adherence to diet and better long-term outcomes of the disease could be achieved with more intense and tailored educational activities involving both patients and parents. Therefore, further studies are also required to examine interventions (e.g. structured transitional care processes) to improve treatment adherence.

## Contributions of individual authors

All authors were involved in recruitment and follow-up of patients and have participated in all aspects of this study.

Alberto Burlina and Chiara Cazzorla were involved in planning of the research.

All authors are responsible for this manuscript, having participated in drafting or revising, and have approved the final submitted version.

## Conflict of interest

Alberto Burlina has received speaker honoraria and travel support from Sanofi Genzyme, Biomarin, Actelion and Nutricia. He is a member of Nutricia and Biomarin European Advisory Board.

Vincenzo Leuzzi has received speaker honoraria and travel support from Biomarin, Actelion, Piam, EryDel and Nutricia. He is a member of Nutricia and Biomarin European Advisory Board.

Filippo Manti has received speaker honoraria and travel support from Biomarin, Piam and Nutricia.

Giulia Bensi, Giacomo Biasucci, Chiara Cazzorla, Antonella Musumeci, Albina Tummolo, Francesco Papadia, Vera Stoppioni, Marcella Vendemiale have no conflicts to declare.

## Informed consent

All procedures followed were in accordance with the ethical standards of the responsible committee on human experimentation (institutional and national) and with the Helsinki Declaration of 1975, as revised in 2000. Informed consent was obtained from all patients for being included in the study.
